# A Case of Malignant Cutaneous Anthrax Localized to the Eyelid

**DOI:** 10.4269/ajtmh.21-1050

**Published:** 2022-02-21

**Authors:** Ayşe Sağmak Tartar, Kutbeddin Demirdağ, Serhat Uysal

**Affiliations:** Department of Infectious Diseases and Clinical Microbiology, Faculty of Medicine, Firat University, Elazığ, Turkey

**Figure 1.**
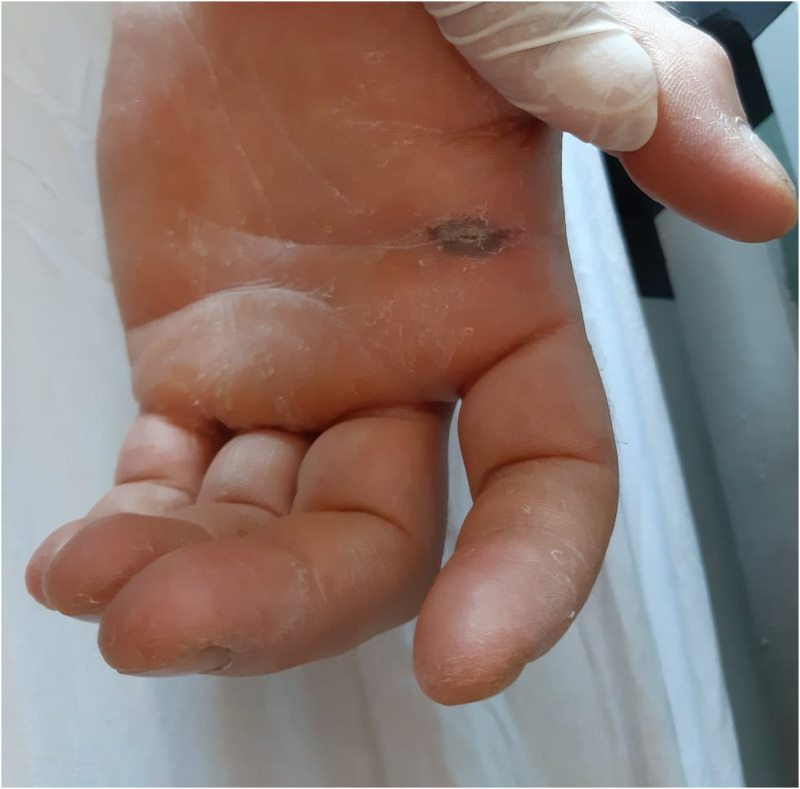
Appearance of the lesion on the patient’s hand. This figure appears in color at www.ajtmh.org.

**Figure 2.**
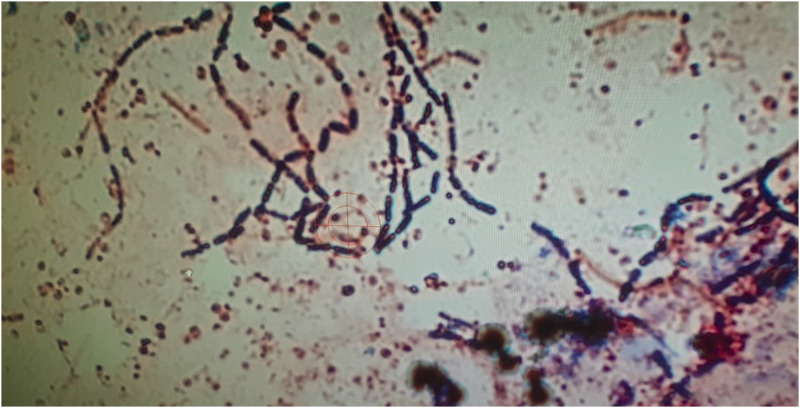
Gram-staining showing gram-positive bacilli in the sample obtained from the eyelid lesion. This figure appears in color at www.ajtmh.org.

A 33-year-old man, prison officer, living in Turkey presented with the complaints of swelling and ache in the right eye. He was admitted to an ophthalmology clinic as inpatient after a preliminary diagnosis of periorbital cellulitis/necrotizing fasciitis. The patient’s medical history was unremarkable. Physical examination revealed 38.2°C fever, and hyperemia and ulcer lesion with a diameter of 2 cm, edema spreading to the face on the right eyelid. In the admission, laboratory findings were as follows: white blood cell: 14,550/mL, neutrophile: 82%, aspartate aminotransferase: 65, C-reactive protein: 112 mg/L, procalcitonin: 0.23. The other parameters were normal. Edema in the right eye progressed within hours. Orbital computed tomography revealed the edema and low-density areas in the right fronto-temporo-occipital region, premaxilla, right parapharyngeal space, and prevertebral fascia. The patient was consulted to infectious disease department. Upon consultation, he was suspected of having anthrax. Epidemiological history revealed that the patient had slaughtered a cow 6 days ago, and a bone had pricked his hand (Figure 1). We collected two swabs for Gram stain and culture. Gram-positive bacilli were noted in the samples obtained from a lesion on his eyelid (Figure 2). Culture drawn did not yield any pathogen. Treatment with meropenem, linezolid, and ciprofloxacin was initiated.

**Figure 3.**
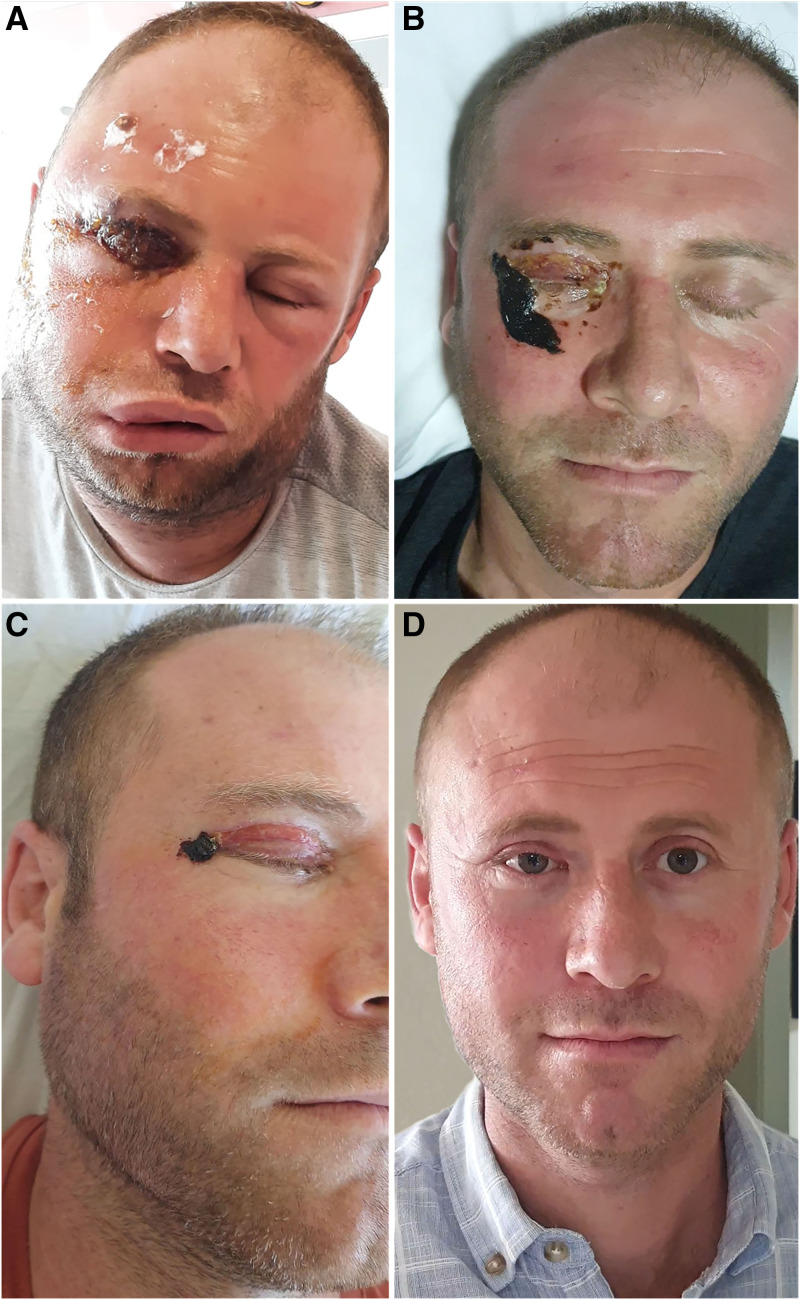
Patient’s lesion on 3 (**A**) and 21 (**B**) and 28 (**C**) and 56 (**D**) days of the follow-up. This figure appears in color at www.ajtmh.org.

The patient developed respiratory distress because of severe edema; therefore, 160 mg/day methylprednisolone was added to the treatment regimen. Consequently, the edema regressed gradually during the follow-up. The treatment was completed in 21 days, and the patient was discharged after a recovery (Figure 3). The patient did not develop visual impairment, but dry eye and eyelid adhesions still persist.

Although the incidence of anthrax has decreased globally and in Turkey, it remains as an endemic disease and is common in regions with widespread uncontrolled animal husbandry. ^1^ The disease is associated with the development of a severe edema in the lesions, particularly on the face and neck region. ^2^ Although there is a lack of controlled studies, steroids can be administered to manage edema-related tracheal obstruction, massive pleural effusion, and massive ascites. ^3^^,^ ^4^ Initial antibacterial therapy should include two bactericidal agents and a protein synthesis inhibitor for cutaneous anthrax with systemic manifestations. ^5^ Considering that the number of anthrax cases decreased, increasing the awareness among physicians will improve the chances of early diagnosis and increase the number of appropriate treatment strategies.
